# Correlation of Utrophin Levels with the Dystrophin Protein Complex and Muscle Fibre Regeneration in Duchenne and Becker Muscular Dystrophy Muscle Biopsies

**DOI:** 10.1371/journal.pone.0150818

**Published:** 2016-03-14

**Authors:** Narinder Janghra, Jennifer E. Morgan, Caroline A. Sewry, Francis X. Wilson, Kay E. Davies, Francesco Muntoni, Jonathon Tinsley

**Affiliations:** 1 Dubowitz Neuromuscular Centre, Molecular Neurosciences Section, Developmental Neurosciences Programme, UCL Institute of Child Health, 30 Guilford Street, London, WC1N1EH, United Kingdom; 2 Summit Therapeutics plc, 85b Park Drive, Milton Park, Abingdon, Oxfordshire, OX14 4RY, United Kingdom; 3 Medical Research Council Functional Genomics Unit, Department of Physiology, Anatomy and Genetics, University of Oxford, Oxford, OX1 3PT, United Kingdom; The Hospital for Sick Children, CANADA

## Abstract

Duchenne muscular dystrophy is a severe and currently incurable progressive neuromuscular condition, caused by mutations in the *DMD* gene that result in the inability to produce dystrophin. Lack of dystrophin leads to loss of muscle fibres and a reduction in muscle mass and function. There is evidence from dystrophin-deficient mouse models that increasing levels of utrophin at the muscle fibre sarcolemma by genetic or pharmacological means significantly reduces the muscular dystrophy pathology. In order to determine the efficacy of utrophin modulators in clinical trials, it is necessary to accurately measure utrophin levels and other biomarkers on a fibre by fibre basis within a biopsy section. Our aim was to develop robust and reproducible staining and imaging protocols to quantify sarcolemmal utrophin levels, sarcolemmal dystrophin complex members and numbers of regenerating fibres within a biopsy section. We quantified sarcolemmal utrophin in mature and regenerating fibres and the percentage of regenerating muscle fibres, in muscle biopsies from Duchenne, the milder Becker muscular dystrophy and controls. Fluorescent immunostaining followed by image analysis was performed to quantify utrophin intensity and β-dystrogylcan and ɣ –sarcoglycan intensity at the sarcolemma. Antibodies to fetal and developmental myosins were used to identify regenerating muscle fibres allowing the accurate calculation of percentage regeneration fibres in the biopsy. Our results indicate that muscle biopsies from Becker muscular dystrophy patients have fewer numbers of regenerating fibres and reduced utrophin intensity compared to muscle biopsies from Duchenne muscular dystrophy patients. Of particular interest, we show for the first time that the percentage of regenerating muscle fibres within the muscle biopsy correlate with the clinical severity of Becker and Duchenne muscular dystrophy patients from whom the biopsy was taken. The ongoing development of these tools to quantify sarcolemmal utrophin and muscle regeneration in muscle biopsies will be invaluable for assessing utrophin modulator activity in future clinical trials.

## Introduction

Duchenne muscular dystrophy (DMD) is a lethal inherited muscle wasting disease caused by mutations in the dystrophin gene that disrupt the open reading frame, preventing production of a functional dystrophin protein [1]. The absence of dystrophin protein from the muscle fibre membrane results in progressive fibre degeneration. A milder allelic form, Becker muscular dystrophy (BMD) is usually caused by in-frame mutations in the dystrophin gene, resulting in the synthesis of reduced levels of a possibly partially functional dystrophin [2,3]. Possible treatment strategies in late stage clinical development include the use of antisense oligonucleotides (AONs) to skip mutated dystrophin exons, allowing the production of a BMD-like dystrophin [4–6]. One drawback of this approach is that the AONs are exon-specific, so one AON is applicable only to a subset of patients carrying specific mutations. Another genetic strategy utilises small molecules to prevent premature protein termination. This approach will allow for a full length dystrophin to be synthesised and will be appropriate for around 10–15% of all DMD patients. Another promising approach in pre-clinical development that has been tested in dystrophin-deficient mice and dogs [7,8], uses adeno-associated virus (AVV) to insert a gene coding for a truncated dystrophin into muscle fibres. However, the major challenges facing AAV gene delivery is the titer necessary to achieve bodywide transfection in the human, the immune reaction against the AAV vector precluding re-administration and how functional the highly truncated dystrophin will be long term. Utrophin, an autosomally-encoded homologue of dystrophin, is first expressed in skeletal muscle during fetal development [9]. Dystrophin and utrophin are both expressed at early gestational stages but by birth in humans utrophin in normal human muscle is confined to the neuromuscular and myotendinous junctions and blood vessels [10]. In dystrophic muscle utrophin is present on regions of fibre repair. [11]. Consequently in some muscular dystrophies, including DMD and BMD, utrophin is present at the muscle fibre sarcolemma due to the significant regeneration taking place, in addition to the up-regulation in DMD and BMD that is present in the absence of dystrophin [9,11]. Utrophin expression is driven by two different promoters, A and B, which regulate the expression of two transcripts utrophin A and B, which have unique expression patterns. The A isoform is expressed in skeletal muscle and present at the sarcolemma of normal fetal muscle fibres, and on both regenerating and mature fibres in DMD and BMD muscle. As with dystrophin, utrophin specifically binds to the costamere in order to stabilize the sarcolemma from contraction-induced damage. Utrophin B is more ubiquitous, being found in most other tissues and cell types [10,12]. Knockdown of utrophin in dystrophin-deficient *mdx* mice causes a considerable worsening of the muscle pathology and function [13,14], suggesting that the presence of utrophin may ameliorate the phenotype of dystrophin-deficient muscles. It also has to be noted that there are some functional differences between dystrophin and utrophin where dystrophin localizes nNOS to the membrane and plays a role in microtubule organization. However there are a number of BMD patients lacking the nNOS binding site in dystrophin, who remain mildly affected and ambulant, suggesting that nNOS tethering at the sarcolemma is not an absolute requirement in order to have a mild phenotype [15,16]. There is therefore considerable interest in utrophin up-regulation as a possible therapeutic option for DMD, as this strategy may have potential for all DMD patients, regardless of their dystrophin mutation. Studies in the *mdx* mouse showed promise with marked reduction in myofibre degeneration and improved phenotype following transgenic up-regulation of utrophin [17]. Further work has demonstrated that a small molecule utrophin modulator, SMT C1100, increased utrophin in *mdx* mice skeletal muscle and diaphragm, leading to a significant decrease in pathology and increased functional benefit [18]. SMT C1100 is currently in clinical development having been shown to be safe and well tolerated in a Phase 1 healthy volunteer study [19]. To determine the efficacy of any therapeutic intervention to up-regulate utrophin, reliable and reproducible quantification of utrophin levels is essential. We have developed a method to quantify sarcolemmal utrophin in frozen muscle biopsies, using methods used previously in our laboratory to quantify intensity of sarcolemmal associated proteins [20]. We quantified utrophin in both mature and regenerating fibres in muscle biopsies from patients with BMD, DMD, carriers of DMD or BMD and non-pathological controls. The dystrophin-associated protein complex (DAPC) complex plays a major role with dystrophin or utrophin to link the extracellular matrix to the actin cytoskeleton. All members of the DAPC are reduced in DMD [21–23] and induction of dystrophin by antisense oligonucleotides leads to increased sarcolemmal expression of DAPC proteins [24,25]. We therefore also investigated whether utrophin plays a role in the stabilisation of members of the DAPC, by correlating the levels of utrophin with β-dystroglycan and ɣ-sarcoglycan levels. If utrophin levels can be maintained at the sarcolemma of a dystrophin-deficient myofibre with a utrophin modulator drug, this should protect the fibre from undergoing necrosis and as a result, reduce muscle regeneration [26]. Therefore we developed a second complementary assay to quantify the percentage number of regenerating fibres in a biopsy, and to help validate the assay we compared the amount of regeneration in BMD and DMD muscle biopsies. Regenerating fibres were identified by staining with antibodies to developmental and fetal myosin as markers of fibre regeneration. Both antibodies were used as a cocktail in order to unequivocally identify all recently regenerating fibres.

## Materials and Methods

### Muscle Biopsies

Sections of skeletal muscle biopsies were obtained from the MRC Centre for Neuromuscular Diseases Biobank London (REC reference number 06/Q0406/33) and partner Eurobiobank. The studies were performed under approval by the NHS National Research Ethics Committee (REC reference number: 05/MRE12/32). All patients or their legal guardians gave written informed consent. Sections from 3 controls, 7 DMD, 3 BMD, 1 BMD/DMD intermediate, one BMD manifesting carrier, one DMD manifesting carrier and 2 DMD non-manifesting carriers were used for this study (Table 1).

**Table 1:** Muscle biopsies used in study.

Patient	Age at biopsy (years)	Phenotype and deletion/mutation	Functional motor score	Age at assessment (years)	Dystrophin levels*
C1	4.6	Muscle biopsy normal	no report	-	+++++
C2	4.6	Mild changes/abnormalities in biopsy Unlikely to have NMD	33/40	4.4	+++++
C3	4.7	Muscle biopsy normal	30/40	5.1	+++++
P1	2.3	BMD manifest. carrier exon 45–47 deletion	40/40	6.9	++++
P2	3.4	DMD manifest. carrier exon 45–50 deletion	36/40	4.3	Positive and negative fibres
P3	4.4	DMD carrier het c.2662G>T (p.Glu888X) exon 21	40/40	12.0	+++++
P4	6.6	DMD carrier het del exon 3–7	49/49^	6.5	Positive and negative fibres
P5	4.2	BMD exon 45–47 deletion	38/40	5.3	++++
P6	10.6	BMD exon 45–49 deletion	37/40	10.6	+++
P7	5.0	BMD exon 45–53 deletion	no report	-	
P8	4.8	BMD/DMD intermediate 45–53 deletion	30/40	5.1	++++
P9	4.8	DMD point mutation c.1388G>A (p.Trp463X) in exon 12	33/40	4.8	-
P10	4.3	DMD exon 10–11 deletion	30/40	4.3	-
P11	4.8	DMD point mutation c.583C>T (p.Arg195X) in exon 7	32/40	4.6	-
P12	7.8	DMD point mutation c.2302 C>T (p.R768X) in exon 19	31/40	8.3	-
P13	8.2	DMD exon 8–11 deletion	34/40	8.2	-
P14	7.3	DMD exon 3–7 deletion	38/40	7.1	++
P15	8.4	DMD point mutation c.9851G>A (p.Trp3284X) in exon 68	23/40	8.3	-

BMD: Becker muscular dystrophy; DMD: Duchenne muscular dystrophy; Manifest: Manifesting, func. motor. score: functional motor score

Dystrophin levels:—complete absence, + severe reduction, ++ moderate reduction, +++ mild reduction, ++++ very mild reduction, +++++ dystrophin levels normal

*Obtained from muscle biopsy report.

^ extended motor score (normal)

### Immunohistochemistry

Frozen transverse muscle sections (7μM) were air dried for 30 minutes and then incubated with a mouse monoclonal antibody to utrophin, (NCL-DRP2 IgG1 Novocastra 1:3 dilution) for 1 hour. Following three 3-minute washes with phosphate buffered saline (PBS), Alexa Flour 488 donkey anti mouse IgG antibody (Molecular Probes A21202 1:100) was applied for 30 minutes. Monoclonal fetal myosin (Novocastra MHCn) and developmental myosin (Novocastra MHCd) antibodies were used as a cocktail at 1:15 and 1:20 respectively for 1 hour following utrophin labelling. A biotinylated anti-mouse antibody (Amersham UK RPN-1001 1:200) was applied for 30 minutes. Sections were then incubated with streptavidin conjugated to Alexa Fluor 594 (Invitrogen S-11227 1:1000) for 15 minutes and mounted in Histomount (National Diagnostics).

Serial sections were stained with a monoclonal antibody to beta- spectrin (Novocastra NCL-SPEC1-CE 1:100) for 1 hour followed by biotinylated anti-mouse antibody and streptavidin conjugated to Alexa Flour A594 as described above.

Sections used for doubling labelling experiments were stained with utrophin, as described above, followed by β-dystroglycan (Novocastra NCL-b-DG IgG2A 1:50) or ɣ-sarcoglycan (Proteintech 18102-1AP Rabbit 1:400) primary antibody for 1 hour. A secondary Alexa Fluor A594 goat anti mouse IgG2A (Molecular Probes A21135 1:100 for 30 minutes) or Alexa Fluor A594 goat anti rabbit IgG1 (Molecular Probes A11012 1:500 for 1 hour) was used following β -dystroglycan and ɣ -sarcoglycan application respectively.

All incubations were carried out at room temperature. Controls, in which the primary antibody was omitted, were performed (S2 Fig). A second control, in which the second primary antibody to β –dystroglycan was omitted, shows that the secondary Alexa Fluor A594 goat anti mouse IgG2A antibody did not cross-react with the mouse IgG1 antibody used to detect utrophin (S3 Fig).

### Intensity measurements

Immunolabelled sections were evaluated using a Leica DMR microscope interfaced to MetaMorph (Molecular Devices, Downingtown, PA, USA). Intensity measurements were obtained using Metamorph software following capture of 4 randomly selected fields, selected out of focus at x20 magnification. An area of stained muscle section taken from an 8 year old DMD patient (P15; Table 1) expressing high levels of utrophin was used to set the exposure times and images of all sections in one experiment were captured at the same time. A control muscle section (C2; Table 1) was used to set ɣ-sarcoglycan and β-dystroglycan exposure times. The intensity was measured by randomly placing 10 regions of interest (ROI) in each image using Metamorph software capturing 40 fibres. If the ROI was in the centre of the fibre, on an area of fibrosis, on a neuromuscular junction, or if more than one measurement per fibre was selected, the region was moved slightly to the nearest fibre membrane. The measured regions included a portion of the cytoplasm and the sarcolemma. In this way, 40 ROI on a total of 40 fibres was captured. No two ROIs were placed on the same muscle fibre. Very small fibres, with a mean diameter of 6μM or less, which were similar in size to capillaries, were excluded from the analysis. Fetal and developmental myosin intensity measurements were taken from the sarcoplasm of fibres as indicators of fibre regeneration using the same method and all intensity measurements were recorded as described previously [20]. Fibre diameter measurements were taken from sections that had been stained with utrophin to delineate fibre membranes, by measuring the diameter of the lesser side of the fibre using Metamorph software [27].

For each region, the minimum intensity value recorded (representative of the cytoplasm or background intensity) was subtracted from the maximum intensity value (which corresponded to the sarcolemma) to correct each measurement for background intensity. To correct for variation of sarcolemmal integrity between samples, we performed the same measurements on serial sections stained with the monoclonal antibody to beta-spectrin. The spectrin intensity values obtained for the control samples were set as the standard to calculate normalization factors.

For each of the antibodies, the minimum intensity value was subtracted from the maximum, then these values (one for each of the 40 fibres analysed) were normalized with the beta-spectrin measurements and plotted on a graph.

There was only background sarcolemmal intensity in sections in which the primary antibodies were omitted (S1 Table; S2 Fig).

### Data analysis

The mean ± standard error of mean (SEM) is represented for normally-distributed data, whilst the median and interquartile range (IQR) is shown for data not normally distributed. A t-test or one-way ANOVA was used to determine differences between groups. Post hoc comparisons were performed with the Tukey test and if data did not meet the assumptions of ANOVA a Kruskal-Wallis one-way ANOVA test was used. Correlations were performed using Pearson’s or Spearman’s test. Statistical analysis was done using Graph Pad Prism and significance level was set at p ≤ 0.05.

## Results

### DMD patient biopsies have significantly higher utrophin levels compared to BMD patients

Intensity quantification methods were used to quantify utrophin on mature muscle fibre sarcolemma in sections of quadriceps muscles (Table 1). Utrophin levels were normalised to spectrin stained on serial sections.

**Fig 1:**
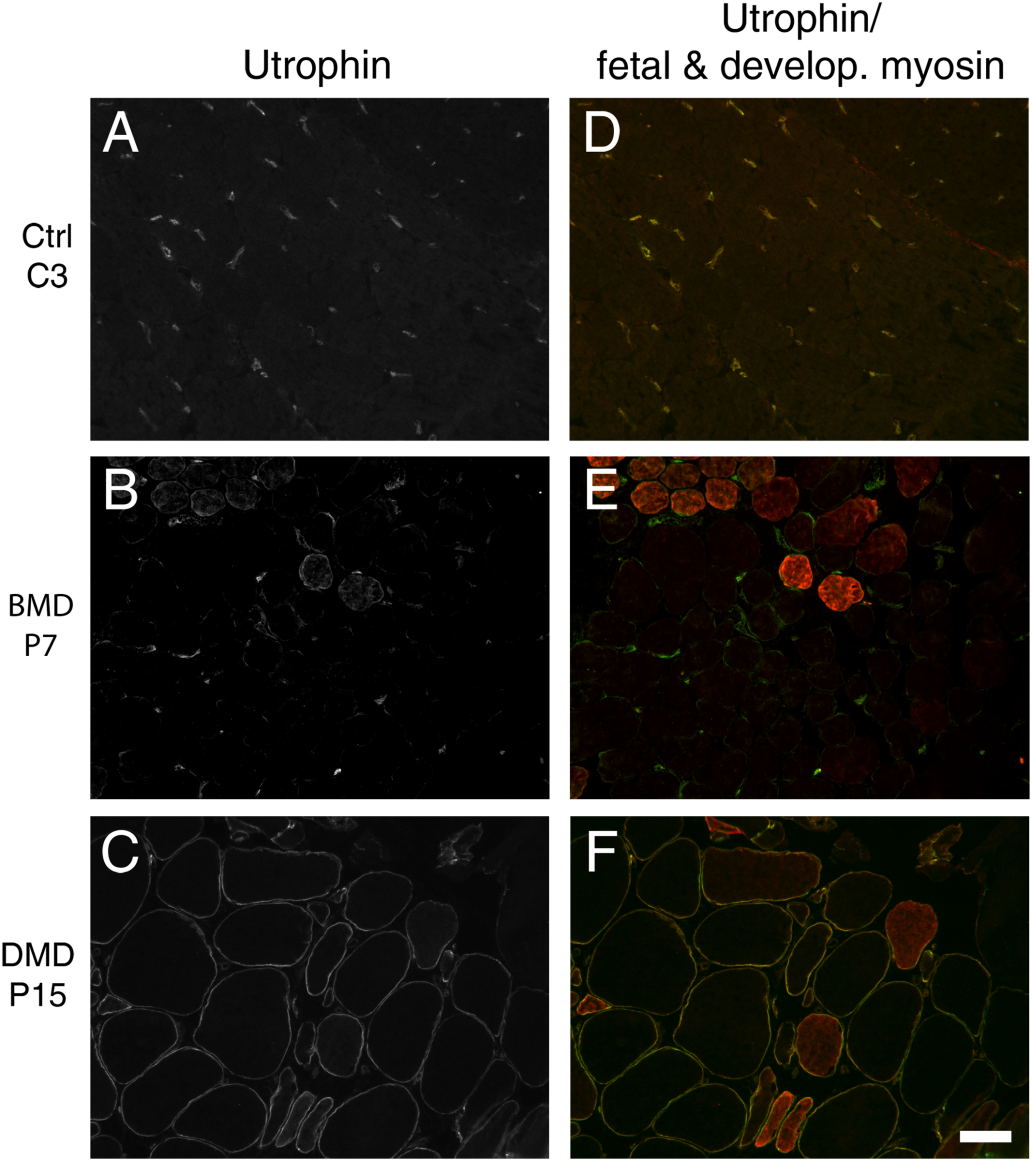
Co-immunostaining of muscle sections with utrophin and fetal and developmental myosin antibodies. (A-C) No sarcolemmal utrophin staining in control (C3), weak labelling in BMD (P7) and strong labelling in DMD (P15) muscle sections used for intensity analysis. (D-F) Utrophin merged with fetal and developmental (develop.) myosin staining to identify regenerating fibres, which were excluded from the analysis in this experiment. White bar—50μM.

**Fig 2:**
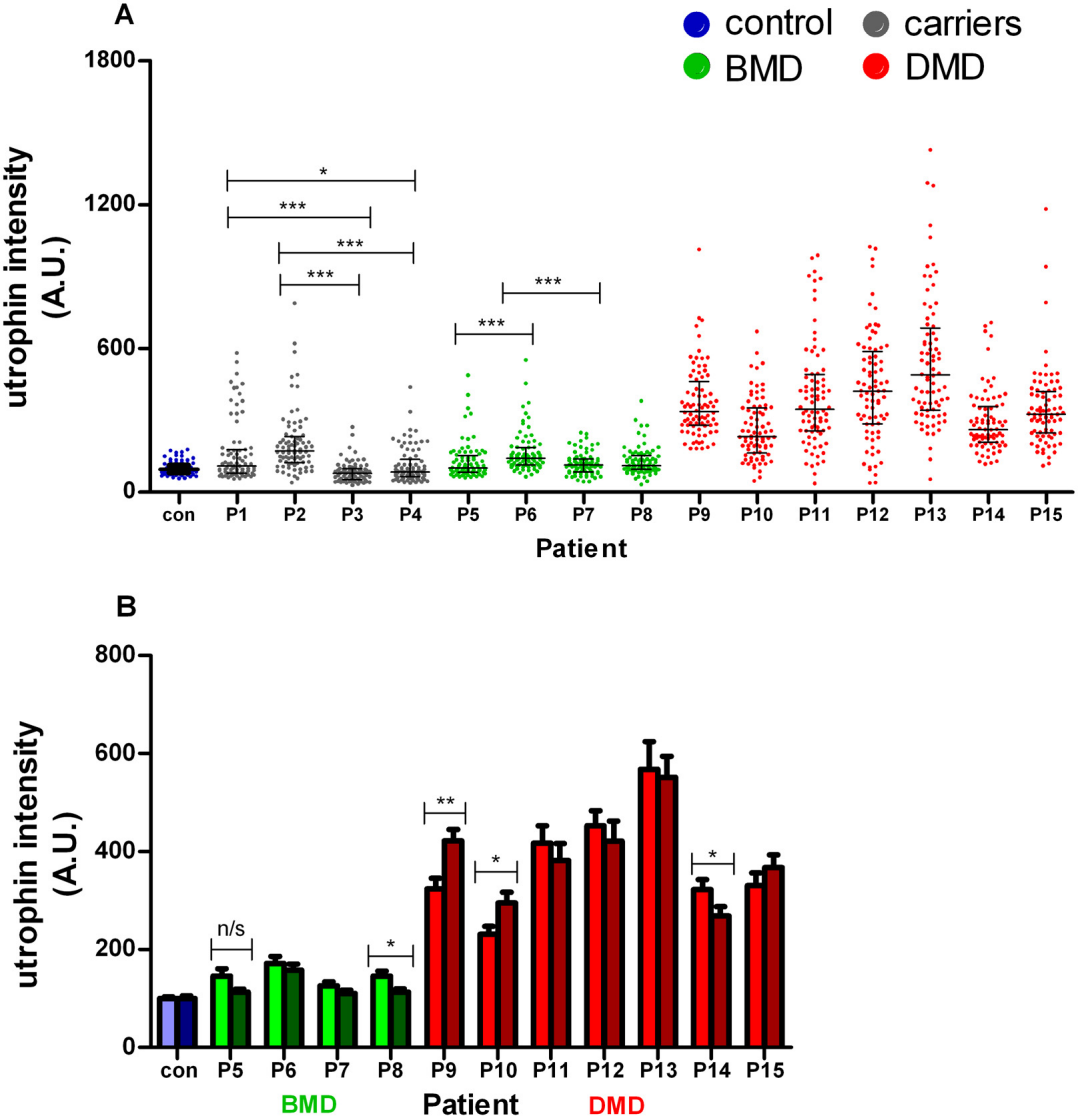
Quantification of utrophin in mature fibres by Metamorph software following normalisation to spectrin. (A) Utrophin intensity (arbitrary units; A.U.) quantified in control (blue dots), carrier (manifesting P1 and P2; non-manifesting P3 and P4; grey dots), BMD (P5-7; green dots), BMD/DMD intermediate (P8; green dots) and DMD (P9-15; red dots) quadriceps muscle. The dot plot depicts the median utrophin intensity (± IQR) in mature muscle fibres from replicate experiments performed on the same muscle but stained and quantified on separate occasions (80 fibres in total analysed). Each point represents a single sarcolemmal intensity reading on one individual myofibre, normalised to spectrin and expressed as a percentage of the average intensity in the 3 non-pathological controls. Intensity measurements distinguish BMD from DMD muscle biopsies. Data are from 2 replicate experiments combined and a Kruskal-Wallis one way analysis of variance followed by Dunn’s test was performed for all comparisons. (B) The variability between replicate experiments performed on control, BMD and DMD muscle biopsies. Wilcoxon matched pair analysis used for comparison between replicate experiments (* p<0.05;** *p*<0.01; *** *p*<0.001; n/s, not significant)

Utrophin intensity was increased on mature muscle fibres in the 7 DMD biopsy samples P9–15 (median: 332; IQR: 238–463) compared to the 3 BMD samples P5–7 (median:119; IQR:91.7–156) (Fig 1) and the single BMD/DMD intermediate patient P8 (median: 111; IQR: –94.5–152) analysed (Fig 2a). The difference in utrophin intensity between the 3 BMD and 7 DMD samples was highly significant for all BMD/DMD comparisons (Kruskal-Wallis followed by Dunn’s test; p<0.0001 for all comparisons except P6 vs P10; p<0.001)).

Utrophin levels were significantly higher in the older BMD patient P6 (median: 141; IQR: 113–186.5) aged 10 years of age, than in the younger BMD patients P5 (median: 101; IQR: 83–153; p< 0.0001) and P7 (median: 113.7; IQR: 83.7–138; Kruskal-Wallis followed by Dunn’s test) aged 4 and 5 years respectively. A similar analysis between these 3 BMD patients and 3 controls revealed significantly greater utrophin intensity in only the older BMD patient P6 (median: 141; IQR: 113–186.5) compared to control muscle (100 ± 3.1), P < 0.0001. A significant increase in utrophin intensity on mature fibres was also evident in the BMD and DMD manifesting carriers P1 (median: 109; IQR: 79–177) and P2 (median: 171; IQR: 122–230.6) compared to the 2 non-manifesting carriers P3 (median: 78; IQR: 52.5–97.7) and P4 (median: 83.6; IQR: 65–137), (Fig 2a). Both non-manifesting carriers, however, showed some fibres with utrophin (Fig 2a). P1, P2 (manifesting) and P4 (non-manifesting) all showed abnormalities in dystrophin expression (Table 1). Utrophin levels in relation to the amount of dystrophin were not examined in the carriers.

To determine reproducibility of the method, utrophin quantification on mature muscle fibres was repeated on serial sections from the same control, BMD and DMD muscles (Fig 2b; Table 2). There was little variability in the measurements from BMD samples between the two experiments and no significant differences between utrophin intensity in each experiment for 4 of the 7 DMD samples. However a Wilcoxon matched-pairs signed rank test revealed significant variability between replicate experiments for the remaining three DMD patients P9 (p = 0.0025), P10 (p = 0.0219) and P14 (p = 0.0417) (Fig 2b; Table 2).

**Table 2:** Normalised utrophin intensity (mean ± standard error of mean).

Patient	Genotype	Utrophin intensity (40 fibres) Exp. 1	utrophin intensity (40 fibres) Exp. 2	Mean comparison (p value)
Control	Normal	*100 ± 3.3	*100 ± 5.3	-
P5	BMD	145.4 ± 15.9	112.8 ± 6.3	-
P6	BMD	171.9 ± 14.6	158.3 ±12.1	-
P7	BMD	126.5 ± 7.9	110.5 ± 6.	-
P8	BMD/DMD	145.5 ± 10.6	113.1 ± 6.8	0.0219
P9	DMD	323 ±22	422.3 ± 22.9	0.0025
P10	DMD	230.8 ± 16.	294.9 ± 22.3	0.0219
P11	DMD	417 ± 36	382 ± 34.4	-
P12	DMD	453.3 ± 30	421.7 ± 40.7	-
P13	DMD	567.8 ± 57	551.9 ± 42.7	-
P14	DMD	322 ± 20.7	268.4 ± 19.4	0.0417
P15	DMD	330.2 ± 26	367.3 ± 26.1	-

* mean utrophin intensity from 3 controls

BMD: Becker muscular dystrophy; DMD: Duchenne muscular dystrophy; Exp.: experiment

Values are mean ± sem.

Mean comparison of 2 replicate experiments with statistical significance shown.

### Regenerating fibres have higher utrophin levels than mature fibres in both BMD and DMD skeletal muscle biopsies

Next, we compared sarcolemmal utrophin levels between mature and regenerating muscle fibres (determined by the presence of developmental and fetal myosin) in sections from 3 BMD patients from 4 to10 years of age (P5, P6 and P7) and 6 DMD patients from 4 to 8 years of age (P9, P10, P11, P12, P14 and P15) by co-immunostaining utrophin with a cocktail of fetal and developmental myosin antibodies. Myosin and utrophin intensity readings and fibre diameter measurements were recorded in fibres that were positive and fibres negative for fetal and developmental myosin in randomly-encountered areas.

**Fig 3:**
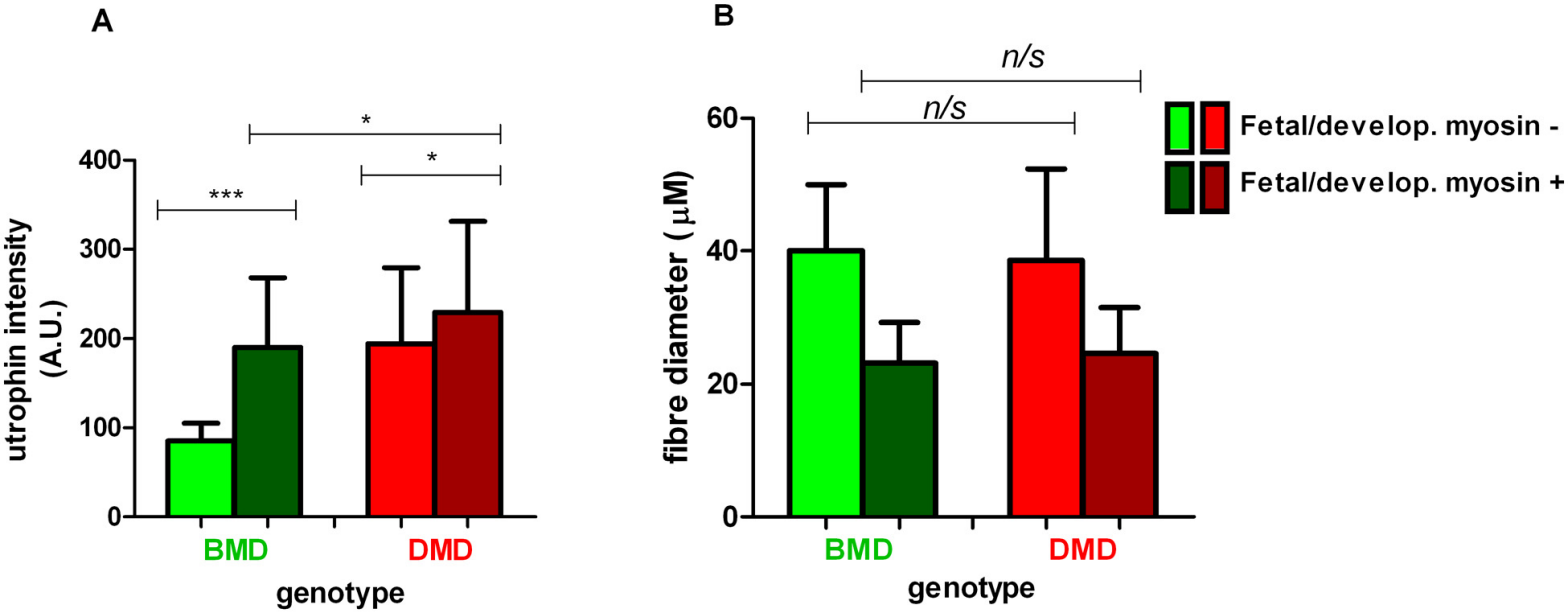
Quantification of utrophin intensity on regenerating fibres in BMD and DMD. (A). Utrophin intensity (arbitrary units; A.U.) and (B) fibre diameter quantified in both fetal and developmental (fetal/develop) myosin negative (light green/red) and positive (dark green/red) muscle fibres in 3 BMD (P5, 6 and 7) and 6 DMD (P9, P10, P11, P12, P14 and P15) (pooled analysis). A single sarcolemmal utrophin measurement was taken from randomly selected fetal and developmental myosin positive and negative fibres from 4 fields of view in areas of muscle regeneration. (n = 39) for P3, (n = 27) for P7, (n = 46) for P8, (n = 60) for P2, (n = 45) for P3, (n = 55) for P4, (n = 53) for P17, (n = 25) for P19 and (n = 49) for P21 where n = total number of fetal/developmental myosin positive and negative fibres analysed for each muscle biopsy. A Kruskal-Wallis one way analysis of variance followed by Dunn’s test was performed (median and IQR depicted; * p<0.05; *** *p*<0.001, n/s, not significant).

A group analysis revealed, as expected, significantly higher utrophin levels on regenerating fibres in BMD muscle, with more than double the utrophin intensity found on the membrane of these fibres (median: 190; IQR: 134–268) compared to mature fibres (median: 85; IQR: 66.5–105; Kruskal-Wallis one way analysis of variance followed by Dunn’s test; Fig 3a). Similarly, in the DMD samples, there was significantly more utrophin localised to the sarcolemma of regenerating fibres (median: 229; IQR: 174–331) than mature muscle fibres (median 194; 138.5–279; p<0.0019 Fig 3a).

Analysis of fibre diameter showed that, as expected, the regenerating fibres were significantly smaller than mature muscle fibres in both BMD and DMD muscle (Fig 3b). However there was no difference in size between regenerating fibres in BMD (median: 23.2; IQR: 19–29 μM) and DMD muscle (median: 24.6; IQR: 17.4–31.5 μM) (Kruskal-Wallis test).

### Stabilisation of ɣ-sarcoglycan and β-dystrogylcan by utrophin in DMD muscle biopsies

**Fig 4:**
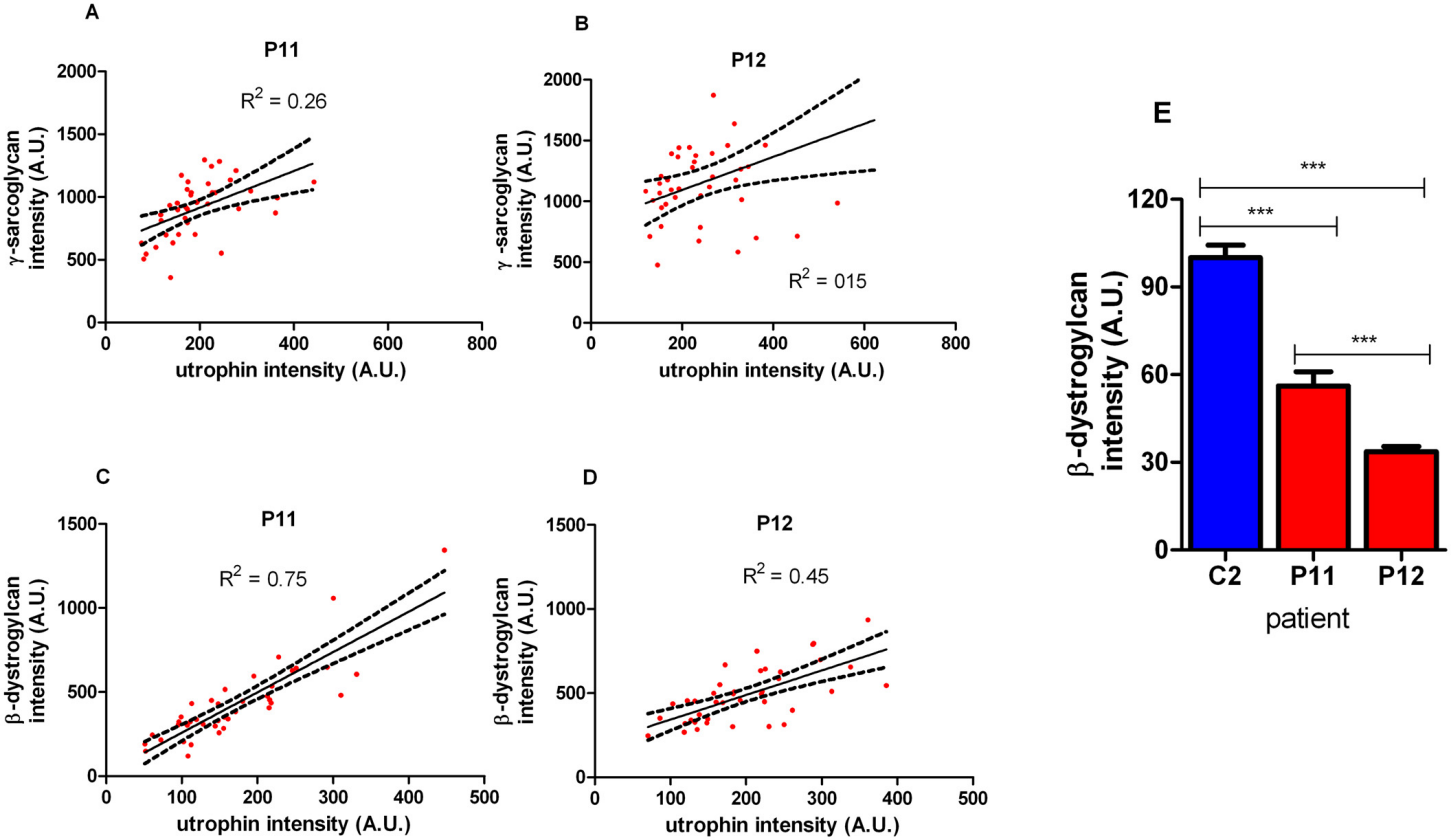
Correlation of utrophin immunostaining with β-dystroglycan and ɣ-sarcoglycan in muscle fibres. (A, B) ɣ-sarcolgycan intensity (arbitrary units; A.U.) on muscle fibres from 4 (P11) and 8 (P12) year old DMD muscle and (C, D) β-dystrogylcan intensity (A.U.) in muscle fibres from 4 (P11) and 8 (P12) year old DMD patients revealed significant correlations with utrophin using Pearson’s and Spearman’s test. Regression line with 95% confidence intervals is shown. (E) The intensity of β-dystroglycan was significantly reduced in DMD P11 and 12 following normalisation to spectrin and expression relative to control muscle biopsy, C2. (One Way ANOVA with Tukey’s: ** *p*<0.01; *** *p*<0.001)

The correlation of utrophin levels with the dystrophin associated protein complex (DAPC) proteins β-dystroglycan (β-DG) and ɣ-sarcoglycan (ɣ-SG) levels was investigated in muscle sections from 1 young (P11; 4 years of age) and 1 older DMD patient (P12; 8 years of age) by co-labelling utrophin with either β-DG or ɣ-SG antibody (S1 Fig). Intensity analysis revealed a significant positive correlation between utrophin levels and ɣ-SG subunit levels in individual fibres in the muscle of the 4 year old DMD patient P11 (Pearson’s, p = 0.0008) but not in the 8 year old P12 (Spearman’s, p = 0.1166; Fig 4a and 4b). There was also a positive correlation between utrophin and β-DG levels in muscle fibres of DMD P11 (Spearman’s, r^2^ = 0.75) and P12 (Pearson’s, r^2^ = 0.45), both of which were highly significant (p<0.0001; Fig 4c and 4d). We quantified the levels of β-DG by normalising to spectrin intensity (Fig 4e). There was a 44% and 66% reduction in β-DG intensity in the sarcolemma of muscle fibres from the young DMD patient (P11) and the older patient P12 compared to the normal control muscle C2 (one way ANOVA with Tukey test; p<0.0001).

### There are significantly more regenerating muscle fibres in DMD than in BMD muscle biopsies

**Fig 5:**
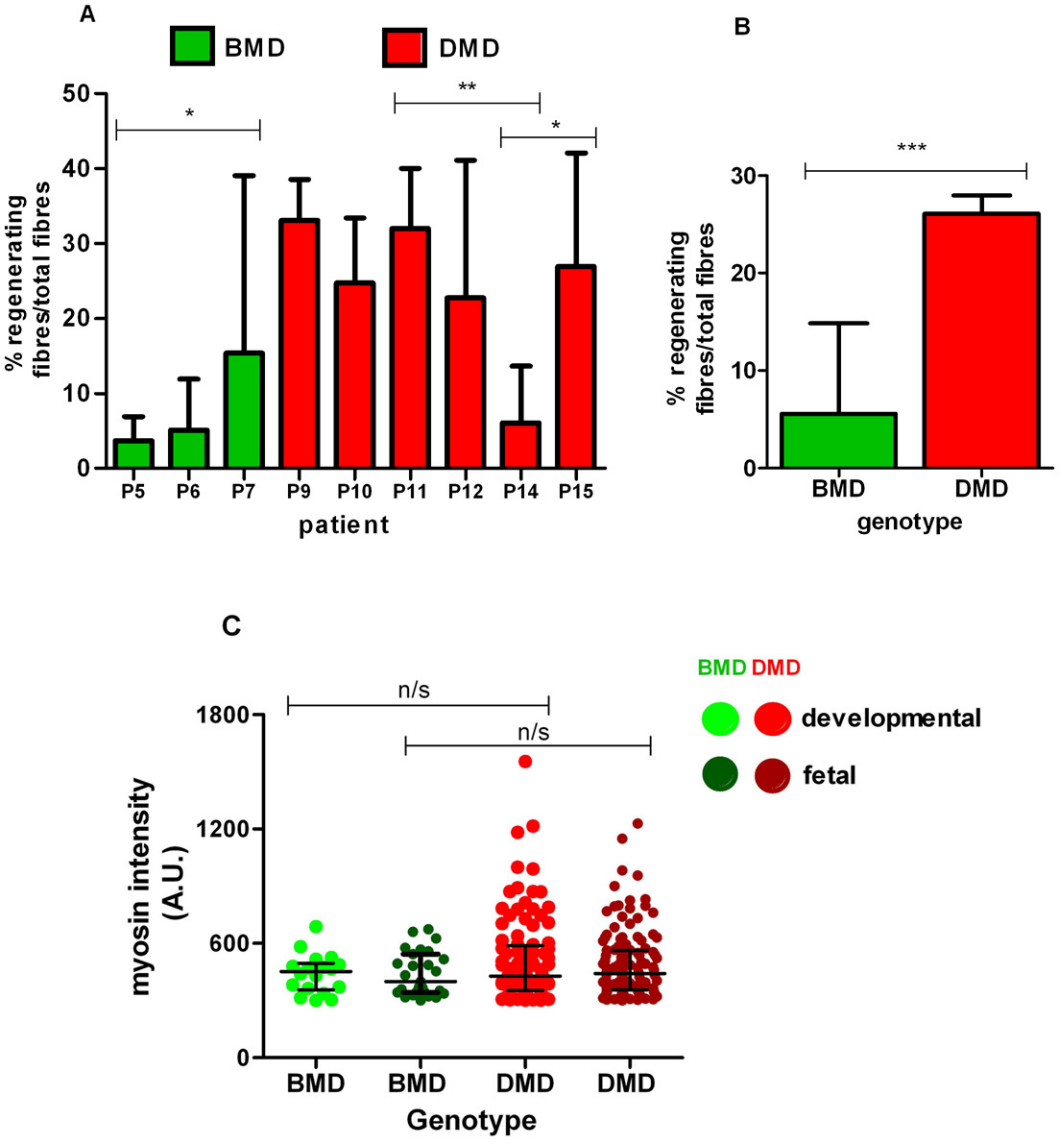
Quantification of regeneration in BMD and DMD. (A) Regenerating myofibres (i.e. fetal and developmental myosin positive), quantified in 3 BMD (green) and 6 DMD (red) muscle biopsies show variable regeneration levels in BMD. Counts obtained from 10 random fields (except P7 and P9 where 9 fields from each were quantified) and expressed as a percentage of the total number of fibres. (B) Pooled analysis of total regenerating fibres showed double the number in DMD compared to BMD and (C) increased mean intensity of both myosins was seen in DMD ((n = 100), developmental; (n = 124), fetal total fibres) compared to BMD ((n = 18), developmental; (n = 27), fetal total fibres analysed) muscle fibres when sections were stained with either fetal or developmental myosin only. No regenerating fibres were present in BMD muscle section P5. Kruskal-Wallis followed by Dunn’s test performed (median and IQR depicted; * p<0.05; ** *p*<0.01; *** *p*<0.001, n/s—not significant).

Muscle regeneration was assessed in the same BMD (P5, 6 and 7) and DMD (P9, 10, 11, 12,14 and 15) sections used to show that regenerating fibres have higher utrophin levels, by quantifying the number of fibres containing both fetal and developmental myosin and expressing this as a percentage of the total number of fibres. The amount of regeneration varied between the 3 BMD muscle biopsies, with P7 having significantly more regenerating fibres (median: 15.4; IQR: 5–39) than BMD P5 (median: 3.7; IQR: 0–6.9), which was significant (Mann-Whitney t-test, p = 0.0194; Fig 5a).

Five of the six DMD muscle biopsies (P9, 10, 11, 12 and 15, aged 4.8, 4.3, 4.8, 7.8 and 8.2 years) analysed had high numbers of regenerating fibres, ranging from 24 to 33% of total muscle fibres in the 10 areas analysed. There were no significant differences in percentages of regenerating fibres between these 5 DMD cases (One way ANOVA with Tukey’s test; Fig 5a). However, DMD muscle biopsy P14 had a lower number of regenerating fibres (median: 6; IQR: 3.1–13.7) when compared to P9 (mean 32.8 ± 3%; p = 0.0036), P10 (mean 28 ± 3.8%; p = 0.0036), P11 (mean 32 ± 3%; p = 0.0058) and P15 (mean 28 ± 3.8%; p = 0.0113) (Fig 5a, Mann-Whitney t test). Further analysis by pooling all the fibre data from the BMD and DMD data showed that there was twice the number of regenerating fibres in DMD (median: 27; IQR: 18.5–36) than in BMD muscles (median: 5.6; IQR: 2.7–14.8), with a statistically significant difference of p<0.0001 (Mann- Whitney t test, Fig 5b).

To determine whether the fetal and developmental myosins were differentially expressed in fibres, serial sections of these BMD and DMD muscles were immuno-labelled with either fetal or developmental myosin antibodies. Although both myosins were expressed at similar mean intensity levels in BMD and DMD muscles, there were a number of fibres with increased intensity of both myosins in DMD (developmental; median: 428; IQR: 353–588, neonatal, median: 441; IQR: 356–564) compared to BMD muscles (developmental; median 453; IQR: 356–495, fetal: median: 399; IQR: 342–544). This difference was not statistically significant (Kruskal-Wallis followed by Dunn’s test, Fig 5c). However, it should be noted that only 2 of the 3 BMD muscle sections (P6 and 7) used for this experiment stained with developmental myosin contained regenerating/positive fibres.

### Correlation between amount of fibre regeneration and functional motor assessment in BMD and DMD

**Fig 6:**
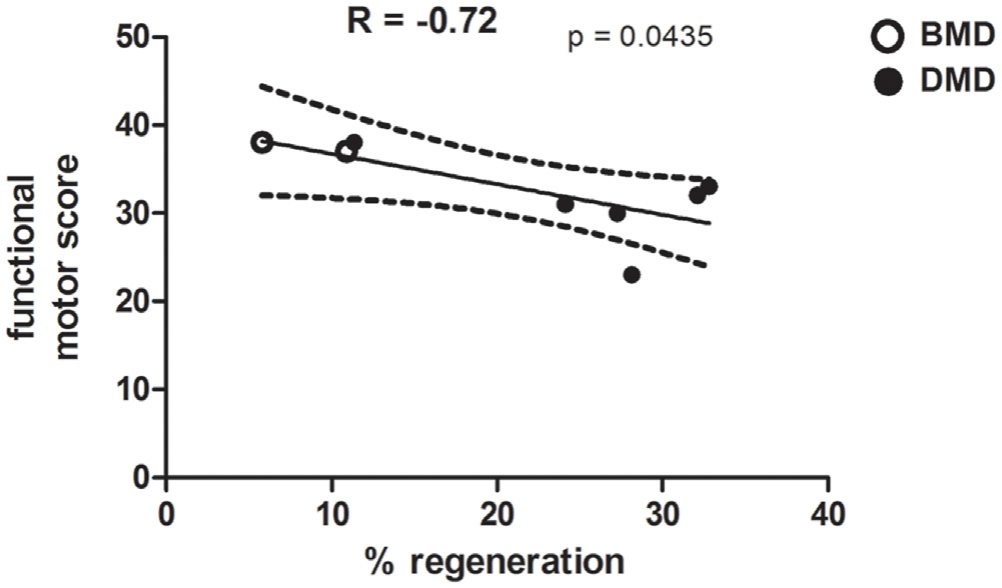
Correlation of the percentage of regenerating fibres with functional motor score in BMD and DMD. Correlation between functional motor score (assessment from a total score of 40) and the level of regeneration in quadriceps muscle from BMD (n = 2) and DMD (n = 6) patients. Dashed lines show 95% confidence intervals.

The percentage of regenerating fibres in BMD (P5 and P6) and DMD (P9, 10, 11, 12, 14 and P15) muscle biopsies were correlated with the patient’s functional motor score (Fig 6). BMD P7 was omitted from the analysis, as no clinical report was available. A Pearson’s test revealed a negative correlation between the level of regeneration and the functional motor score, with a lower motor score evident with an increasing percentage of regenerating fibres within the muscle biopsy, which was significant (p = 0.0435; Fig 6).

## Discussion

A therapeutic strategy that is being pursued in DMD is the modulation of utrophin in dystrophin-deficient muscle fibres, i.e. the re-programming of utrophin transcription such that utrophin RNA and protein is continually expressed in mature fibres. The replacement of dystrophin with utrophin has been shown to be effective following transgenic and pharmacological approaches to increase utrophin in *mdx* mice [18,28].

For therapeutic efficacy, sufficient utrophin linked to the DAPC must be present at the sarcolemma of muscle fibres to prevent or slow the fibres from undergoing necrosis. To determine this, accurate quantification of utrophin and components of the DAPC at the muscle fibre sarcolemma needs to be demonstrated. If utrophin modulation results in more utrophin localised to the sarcolemma in mature dystrophin deficient fibres for longer, then a reduction in muscle fibre necrosis would be predicted.

To this end, we have developed protocols to quantify sarcolemmal utrophin and members of the DAPC using a semi-quantitative immunocytochemical technique originally developed to quantify dystrophin [20]. A novel protocol has been developed immunostaining fetal and developmental myosins in the sarcoplasm of muscle fibres to quantify the percentage of regenerating fibres in a muscle biopsy (Fig 2). Utrophin quantification was reproducible between experiments (Fig 2b).

We found, as expected, increased numbers of mature muscle fibres with utrophin in DMD compared to BMD [29] and control muscle biopsies [30] (Fig 1a). There are lower levels of utrophin in BMD compared to DMD mature muscle fibres, as the former express some dystrophin that would be expected to influence the level of utrophin.

There is a significant difference between utrophin levels in regenerating and non-regenerating DMD muscle fibres (Fig 3). The difference between the situation in fibre repair and the utrophin therapeutic approach is where the utrophin is localised. In DMD, the transverse section identifies the fibres that are regenerating by virtue of the utrophin staining. However this is a very local event where the damaged membrane is being degraded and repaired via myoblast fusion. If one were to travel further along that fibre, one would find regions that have not started the regenerative process. This intermittent expression of utrophin along the fibre will have limited effect on protecting the fibre and the muscle. The therapeutic approach is to modulate normal utrophin transcription such that utrophin is present along the whole length of the fibre with no regions without utrophin at the membrane. Indeed this was achieved in the original utrophin transgenic studies where utrophin was ubiquitously expressed in muscle and associated with functional benefit [18]. Compounds developed by Summit to modulate utrophin in the *mdx* mouse have been shown to produce uniform continual staining along the length of the fibres and this is associated with clinical benefit. In contrast, in untreated *mdx* muscle there is punctate staining where it is presumed focussed repair of the fibre is taking place [31].

There was a significant positive correlation of utrophin intensity with β-dystroglycan (β-DG) intensity at the sarcolemma of muscle fibres in a 4 and an 8 year old DMD patient (P11 and P12), but ɣ-sarcoglycan (ɣ-SG) and utrophin intensities had a significantly positive correlation only in fibres from P11, not P12 (Fig 4c and 4d). We also quantified the levels of β-DG by normalising to spectrin intensity in sections of the same muscles. β-DG intensity in fibres in the older P12 was significantly lower than in the younger P11, but whether this is due to the patient age, or other factors, is not known. In both these 2 DMD patients that lack dystrophin (but have utrophin), β-DG intensity was less than in control muscles that have normal dystrophin (but no utrophin). This is not surprising, as there are functional differences between dystrophin and utrophin. Dystrophin binds actin via two separate contact sites whereas utrophin interacts with actin through a single continuous unit (for a review, see [32]). Furthermore, utrophin shows a twofold lower affinity for β-dystroglycan [33]. Nevertheless, both proteins are fully capable of interacting with F-actin and β-dystroglycan, and each alone is sufficient to preserve the sarcolemmal integrity. But dystrophin can recruit nNOS to the sarcolemma, whereas utrophin cannot [34]. In addition, dystrophin binds microtubules with high affinity and pauses microtubule polymerization, whereas utrophin has no activity in either assay [35]. Interestingly, some β-DG remains at the sarcolemma of muscle fibres in the absence of utrophin and dystrophin [36].

The amount of ɣ-SG and β-DG correlated with the level of utrophin in muscle fibres of some DMD patients, indicating that utrophin is able to recruit components of the DAPC at the sarcolemma of dystrophin-deficient muscle fibres (Fig 4). This provides further support of the advantageous effects of utrophin modulation in DMD patients in that pharmacologically maintaining utrophin levels at the costameric binding sites in fibres should also allow for conservation or localisation of the DAPC. This is the first time that the intensity of utrophin and members of the DAPC complex have been quantified simultaneously and significant correlations demonstrated.

The identification of regenerating muscle fibres is not straightforward. Although they can be distinguished from mature muscle fibres on the basis of their size and expression of developmental myosins, there is no single marker that unequivocally identifies a regenerating fibre. Some developmental myosins may be present in muscle fibres that are not regenerating, for example in denervated muscle fibres, and the time after the onset of regeneration affects the expression pattern [37,38]. Embryonic and fetal myosin heavy chains are expressed sequentially in muscle development [39]. They are also expressed in regenerating muscle fibres from approximately 2–3 days after injury and persist for 2–3 weeks [39]. The antibodies that we employed recognise a myosin heavy chain present during fetal development but down regulated by birth (MHCd: developmental myosin antibody) and a myosin present during the first few months after birth in humans (MHCn: neonatal myosin antibody) (unpublished observations). Regenerating fibres express variable levels of both proteins, depending on the stage of regeneration. In dystrophic muscle, the MHCn antibody often detects more fibres than MHCd, which recognises fibres at an earlier stage of maturation. Hence, we chose to use a combination of fetal and embryonic myosin antibodies to identify regenerating fibres at all stages of maturation. Using this antibody combination, we found, as expected [27] a significantly higher percentage of regenerating muscle fibres in DMD biopsy sections compared to BMD biopsies. We also confirmed that regenerating or recently regenerated fibres have increased sarcolemmal utrophin levels [11,40–42].

Interestingly, the percentage of regenerating fibres in BMD and DMD muscle biopsies correlated inversely with the patient’s functional motor score [43], suggesting that muscles undergoing less degeneration and regeneration are more functional. However, the interval between the age at which the biopsy was done and the age at which muscle functional tests were performed (Table 1) will have an effect. However, our sample numbers are small; in addition, it is known that the patient age and DMD genotype, neither of which we have taken into account in our analysis, have an effect on the motor function [44]. Other factors, including the number of surviving myofibres and extent of fibrosis, will also affect muscle function. Similarly, a significant relationship between muscle regeneration and ankle dorsiflexion force in limb girdle muscular dystrophy type 2I (LGMD2I) patients has been demonstrated, but in that study only neonatal myosin was used to distinguish the regenerating fibres [38]. The controls we used had morphologically normal muscle, although the patients had been referred to our neuromuscular service for a clinical reason.

Kleopa et al. [45] quantified utrophin on Western blot and compared the ratio of utrophin:myosin in DMD muscle samples to the ratio of utrophin:myosin in a normal human fetal muscle sample, in which utrophin expression is high. Using this measure, the mean utrophin in DMD was 55% of that in normal fetal muscle, but there was a very large standard deviation. The significance of utrophin levels and severity of disease has been highlighted recently [46] utilizing semi-quantitative western blots to demonstrate similar levels of utrophin protein in severe and less severe DMD samples. The downside to this approach is that any difference in levels of regeneration across the whole biopsy are missed and it is this subtlety that may correlate with disease severity. The development of the quantitative imaging methods described in our manuscript is to remove this variability of total protein quantification approaches with absolute quantification of utrophin levels at the membrane of each fibre. This gives novel insights into the biology of utrophin in regenerating and non-regenerating fibres and the interaction of utrophin with components of the dystroglycan complex in human muscle.

This is a proof of principle study to show that it is possible to quantify differences between DMD and BMD patient biopsies. Future clinical trials would require high throughput methods, as the method we describe is labour-intensive. Consequently the next steps in the further development of these protocols is to develop solutions where the whole biopsy section is scanned and data generated using automated imaging technologies such as those recently used for the quantification of dystrophin [47].

## Conclusions

The tools that we have developed to quantify sarcolemmal utrophin, DAPC proteins and muscle regeneration in muscle biopsies have led to interesting observations correlating levels of utrophin and members of the DAPC and percentage of regenerating fibres to muscle function. These tools will be invaluable for confirming utrophin modulator activity in future clinical trials.

## Supporting Information

S1 Fig Representative transverse sections of P11 muscle stained with antibodies to utrophin and gamma sarcoglycan (A and C) and utrophin and beta dystroglycan (B and D). Scale bar = 50μM.
(TIFF)

S2 Fig Sections stained with secondary antibodies have background sarcolemmal intensities. A. Serial sections of P9 and P11 were labelled with either the secondary antibody used for utrophin immunostaining only, or with a combination of both secondary antibodies used for fetal myosins and utrophin immunostaining. Images were captured for sarcolemmal intensity measurement in 20 random fibres, using the same exposure times used for utrophin intensity measurements. There was no difference in mean sarcolemmal intensity between the sections stained with one or both secondary antibodies for both P9 (unpaired t-test; p = 0.58) and P11 (unpaired t-test; p = 0.32). B. Serial sections of P11 were stained with either (1) Secondary antibody for utrophin only (2) secondary antibodies for utrophin and fetal myosins (3) secondary antibodies for utrophin and gamma sarcoglycan (g-SG) (4) secondary antibodies for utrophin and beta-dystroglycan (b-DG) and images captured for sarcolemmal intensity measurement. 1 and 2: 20 fibres analysed for each; 3 and 4: 10 fibres analysed for each. There were no differences in sarcolemmal intensity between the 4 groups (1 way ANOVA with Tukey’s test). C. Serial sections of P12 were stained with secondary antibodies for (1) utrophin and g-SG, or (2) utrophin and b-DG and images captured for sarcolemmal intensity analysis. 10 fibres were quantified for each combination. There was no significant difference between sarcolemmal intensity between the two groups (unpaired t-test; p = 0.32). The sarcolemmal intensities when only secondary antibodies had been applied were similar to the mean utrophin intensity levels in control muscles (38 ± 1.7; Fig 2).
(TIFF)

S3 Fig The secondary antibody used to detect beta-dystroglycan does not identify utrophin. Section of P12 was stained with mouse monoclonal antibody to utrophin, (NCL-DRP2 IgG1), followed by Alexa Fluor 488 donkey anti mouse antibody (Molecular Probes A21202, anti IgG) and the secondary antibody that was used to detect beta-dystroglycan (Alexa Fluor A594 goat anti mouse IgG2A (Molecular Probes A21135). A. Fibres expressing utrophin, detected by Alexa Flour 488 donkey anti mouse IgG antibody. B. Alexa Fluor A594 goat anti mouse IgG2A antibody does not cross-react with utrophin. C. Merged image of A and B. Scale bar = 50μM.
(TIFF)

S1 Table Sarcolemmal intensity measurements after application of secondary and tertiary antibodies. (DOCX)

## References

[1] Muntoni F, Torelli S, Ferlini A (2003) Dystrophin and mutations: one gene, several proteins, multiple phenotypes. Lancet Neurol 2: 731–740. 14636778 10.1016/s1474-4422(03)00585-4

[2] Chelly J, Gilgenkrantz H, Lambert M, Hamard G, Chafey P, Recan D, et al (1990) Effect of dystrophin gene deletions on mRNA levels and processing in Duchenne and Becker muscular dystrophies. Cell 63: 1239–1248. 2261642 10.1016/0092-8674(90)90419-f

[3] Arahata K, Beggs AH, Honda H, Ito S, Ishiura S, Tsukahara T, et al (1991) Preservation of the C-terminus of dystrophin molecule in the skeletal muscle from Becker muscular dystrophy. J Neurol Sci 101: 148–156. 2033400 10.1016/0022-510x(91)90039-a

[4] Mendell JR, Rodino-Klapac LR, Sahenk Z, Roush K, Bird L, Lowes LP, et al (2013) Eteplirsen for the treatment of Duchenne muscular dystrophy. Ann Neurol 74: 637–647. 10.1002/ana.23982 23907995

[5] Mercuri E, Muntoni F (2013) Muscular dystrophy: new challenges and review of the current clinical trials. Curr Opin Pediatr 25: 701–707. 10.1097/MOP.0b013e328365ace5 24240289

[6] Koo T, Wood MJ (2013) Clinical trials using antisense oligonucleotides in duchenne muscular dystrophy. Hum Gene Ther 24: 479–488. 10.1089/hum.2012.234 23521559

[7] Yoshimura M, Sakamoto M, Ikemoto M, Mochizuki Y, Yuasa K, Miyagoe-Suzuki Y, et al (2004) AAV vector-mediated microdystrophin expression in a relatively small percentage of mdx myofibers improved the mdx phenotype. Mol Ther 10: 821–828. 15509500 10.1016/j.ymthe.2004.07.025

[8] Wang Z, Kuhr CS, Allen JM, Blankinship M, Gregorevic P, Chamberlain JS, et al (2007) Sustained AAV-mediated dystrophin expression in a canine model of Duchenne muscular dystrophy with a brief course of immunosuppression. Molecular therapy: the journal of the American Society of Gene Therapy 15: 1160–1166. 17426713 10.1038/sj.mt.6300161

[9] Clerk A, Morris GE, Dubowitz V, Davies KE, Sewry CA (1993) Dystrophin-related protein, utrophin, in normal and dystrophic human fetal skeletal muscle. Histochem J 25: 554–561. 8407365

[10] Sewry CA, Nowak KJ, Ehmsen JT, Davies KE (2005) A and B utrophin in human muscle and sarcolemmal A-utrophin associated with tumours. Neuromuscul Disord 15: 779–785. 16198105 10.1016/j.nmd.2005.08.002

[11] Helliwell TR, Man NT, Morris GE, Davies KE (1992) The dystrophin-related protein, utrophin, is expressed on the sarcolemma of regenerating human skeletal muscle fibres in dystrophies and inflammatory myopathies. Neuromuscul Disord 2: 177–184. 1483043 10.1016/0960-8966(92)90004-p

[12] Weir AP, Burton EA, Harrod G, Davies KE (2002) A- and B-utrophin have different expression patterns and are differentially up-regulated in mdx muscle. J Biol Chem 277: 45285–45290. 12235137 10.1074/jbc.M205177200

[13] Deconinck N, Rafael JA, Beckers-Bleukx G, Kahn D, Deconinck AE, Davies KE, et al (1998) Consequences of the combined deficiency in dystrophin and utrophin on the mechanical properties and myosin composition of some limb and respiratory muscles of the mouse. Neuromuscul Disord 8: 362–370. 9713852 10.1016/s0960-8966(98)00048-0

[14] van Putten M, Kumar D, Hulsker M, Hoogaars WM, Plomp JJ, van Opstal A, et al (2012) Comparison of skeletal muscle pathology and motor function of dystrophin and utrophin deficient mouse strains. Neuromuscul Disord 22: 406–417. 10.1016/j.nmd.2011.10.011 22284942

[15] Torelli S, Brown SC, Jimenez-Mallebrera C, Feng L, Muntoni F, Sewry CA (2004) Absence of neuronal nitric oxide synthase (nNOS) as a pathological marker for the diagnosis of Becker muscular dystrophy with rod domain deletions. Neuropathol Appl Neurobiol 30: 540–545. 15488030 10.1111/j.1365-2990.2004.00561.x

[16] Anthony K, Cirak S, Torelli S, Tasca G, Feng L, Arechavala-Gomeza V, et al (2011) Dystrophin quantification and clinical correlations in Becker muscular dystrophy: implications for clinical trials. Brain: a journal of neurology 134: 3547–3559. 22102647 10.1093/brain/awr291 PMC3235564

[17] Tinsley JM, Potter AC, Phelps SR, Fisher R, Trickett JI, Davies KE (1996) Amelioration of the dystrophic phenotype of mdx mice using a truncated utrophin transgene. Nature 384: 349–353. 8934518 10.1038/384349a0

[18] Tinsley J, Deconinck N, Fisher R, Kahn D, Phelps S, Gillis JM, et al (1998) Expression of full-length utrophin prevents muscular dystrophy in mdx mice. Nat Med 4: 1441–1444. 9846586 10.1038/4033

[19] Tinsley J, Robinson N, Davies KE (2015) Safety, tolerability, and pharmacokinetics of SMT C1100, a 2-arylbenzoxazole utrophin modulator, following single- and multiple-dose administration to healthy male adult volunteers. J Clin Pharmacol. 10.1002/jcph.468 PMC5024067 25651188

[20] Arechavala-Gomeza V, Kinali M, Feng L, Brown SC, Sewry C, Morgan JE, et al (2010) Immunohistological intensity measurements as a tool to assess sarcolemma-associated protein expression. Neuropathology and applied neurobiology 36: 265–274. 10.1111/j.1365-2990.2009.01056.x 20002311

[21] Matsumura K, Tome FM, Ionasescu V, Ervasti JM, Anderson RD, Romero NB, et al (1993) Deficiency of dystrophin-associated proteins in Duchenne muscular dystrophy patients lacking COOH-terminal domains of dystrophin. J Clin Invest 92: 866–871. 8349821 10.1172/JCI116661 PMC294925

[22] Ohlendieck K, Matsumura K, Ionasescu VV, Towbin JA, Bosch EP, Weinstein SL, et al (1993) Duchenne muscular dystrophy: deficiency of dystrophin-associated proteins in the sarcolemma. Neurology 43: 795–800. 8469343 10.1212/wnl.43.4.795

[23] Mizuno Y, Yoshida M, Nonaka I, Hirai S, Ozawa E (1994) Expression of utrophin (dystrophin-related protein) and dystrophin-associated glycoproteins in muscles from patients with Duchenne muscular dystrophy. Muscle Nerve 17: 206–216. 8114791 10.1002/mus.880170212

[24] Cirak S, Arechavala-Gomeza V, Guglieri M, Feng L, Torelli S, Anthony K, et al (2011) Exon skipping and dystrophin restoration in patients with Duchenne muscular dystrophy after systemic phosphorodiamidate morpholino oligomer treatment: an open-label, phase 2, dose-escalation study. Lancet. 10.1016/S0140-6736(11)60756-3 PMC3156980 21784508

[25] Cirak S, Feng L, Anthony K, Arechavala-Gomeza V, Torelli S, Sewry C, et al (2012) Restoration of the dystrophin-associated glycoprotein complex after exon skipping therapy in Duchenne muscular dystrophy. Molecular therapy: the journal of the American Society of Gene Therapy 20: 462–467. 22086232 10.1038/mt.2011.248 PMC3277241

[26] Squire S, Raymackers JM, Vandebrouck C, Potter A, Tinsley J, Fisher R, et al (2002) Prevention of pathology in mdx mice by expression of utrophin: analysis using an inducible transgenic expression system. Hum Mol Genet 11: 3333–3344. 12471059 10.1093/hmg/11.26.3333

[27] Dubowitz V, Sewry CA and Oldfors A 2013 Muscle Biopsy: A Practical Approach 4th Edn Elsevier, Oxford

[28] Tinsley JM, Fairclough RJ, Storer R, Wilkes FJ, Potter AC, Squire SE, et al (2011) Daily treatment with SMTC1100, a novel small molecule utrophin upregulator, dramatically reduces the dystrophic symptoms in the mdx mouse. PLoS One 6: e19189 10.1371/journal.pone.0019189 21573153 PMC3089598

[29] Taylor J, Muntoni F, Dubowitz V, Sewry CA (1997) The abnormal expression of utrophin in Duchenne and Becker muscular dystrophy is age related. Neuropathol Appl Neurobiol 23: 399–405. 9364465

[30] Vainzof M, Passos-Bueno MR, Man N, Zatz M (1995) Absence of correlation between utrophin localization and quantity and the clinical severity in Duchenne/Becker dystrophies. Am J Med Genet 58: 305–309. 8533838 10.1002/ajmg.1320580403

[31] Guiraud S, Squire SE, Edwards B, Chen H, Burns DT, Shah N, et al (2015) Second-generation compound for the modulation of utrophin in the therapy of DMD. Hum Mol Genet 24: 4212–4224. 10.1093/hmg/ddv154 25935002 PMC4492389

[32] Ervasti JM (2007) Dystrophin, its interactions with other proteins, and implications for muscular dystrophy. Biochim Biophys Acta 1772: 108–117. 16829057 10.1016/j.bbadis.2006.05.010

[33] Ishikawa-Sakurai M, Yoshida M, Imamura M, Davies KE, Ozawa E (2004) ZZ domain is essentially required for the physiological binding of dystrophin and utrophin to beta-dystroglycan. Hum Mol Genet 13: 693–702. 14962982 10.1093/hmg/ddh087

[34] Li D, Bareja A, Judge L, Yue Y, Lai Y, Fairclough R, et al (2010) Sarcolemmal nNOS anchoring reveals a qualitative difference between dystrophin and utrophin. J Cell Sci 123: 2008–2013. 10.1242/jcs.064808 20483958 PMC2880012

[35] Belanto JJ, Mader TL, Eckhoff MD, Strandjord DM, Banks GB, Gardner MK, et al (2014) Microtubule binding distinguishes dystrophin from utrophin. Proc Natl Acad Sci U S A 111: 5723–5728. 10.1073/pnas.1323842111 24706788 PMC3992671

[36] Johnson EK, Zhang L, Adams ME, Phillips A, Freitas MA, Froehner SC et al (2012) Proteomic analysis reveals new cardiac-specific dystrophin-associated proteins. PLoS One 7: e43515 10.1371/journal.pone.0043515 22937058 PMC3427372

[37] Gross JG, Morgan JE (1999) Muscle precursor cells injected into irradiated mdx mouse muscle persist after serial injury. Muscle Nerve 22: 174–185. 10024130 10.1002/(sici)1097-4598(199902)22:2<174::aid-mus5>3.0.co;2-s

[38] Krag TO, Hauerslev S, Sveen ML, Schwartz M, Vissing J (2011) Level of muscle regeneration in limb-girdle muscular dystrophy type 2I relates to genotype and clinical severity. Skelet Muscle 1: 31 10.1186/2044-5040-1-31 21970816 PMC3197566

[39] Schiaffino S, Rossi AC, Smerdu V, Leinwand LA, Reggiani C (2015) Developmental myosins: expression patterns and functional significance. Skelet Muscle 5: 22 10.1186/s13395-015-0046-6 26180627 PMC4502549

[40] Tachi N, Watanabe Y, Ohya K, Chiba S (1993) Asymptomatic Becker muscular dystrophy: histological changes in biopsied muscles. Acta Paediatr Jpn 35: 409–411. 8256625 10.1111/j.1442-200x.1993.tb03082.x

[41] Wilson LA, Cooper BJ, Dux L, Dubowitz V, Sewry CA (1994) Expression of utrophin (dystrophin-related protein) during regeneration and maturation of skeletal muscle in canine X-linked muscular dystrophy. Neuropathol Appl Neurobiol 20: 359–367. 7808586 10.1111/j.1365-2990.1994.tb00981.x

[42] Lin S, Gaschen F, Burgunder JM (1998) Utrophin is a regeneration-associated protein transiently present at the sarcolemma of regenerating skeletal muscle fibers in dystrophin-deficient hypertrophic feline muscular dystrophy. J Neuropathol Exp Neurol 57: 780–790. 9720493 10.1097/00005072-199808000-00007

[43] Scott OM, Hyde SA, Goddard C, Dubowitz V (1982) Quantitation of muscle function in children: a prospective study in Duchenne muscular dystrophy. Muscle Nerve 5: 291–301. 7099196 10.1002/mus.880050405

[44] Ricotti V, Ridout DA, Pane M, Main M, Mayhew A, Mercuri E, et al (2015) The NorthStar Ambulatory Assessment in Duchenne muscular dystrophy: considerations for the design of clinical trials. J Neurol Neurosurg Psychiatry. 10.1136/jnnp-2014-309405 PMC4752678 25733532

[45] Kleopa KA, Drousiotou A, Mavrikiou E, Ormiston A, Kyriakides T (2006) Naturally occurring utrophin correlates with disease severity in Duchenne muscular dystrophy. Hum Mol Genet 15: 1623–1628. 16595608 10.1093/hmg/ddl083

[46] Vainzof M, Feitosa L, Canovas M, Ayub-Guerrieri D, Pavanello RC, Zatz M (2016) Concordant utrophin upregulation in phenotypically discordant DMD/BMD brothers. Neuromuscul Disord. 10.1016/j.nmd.2016.01.001 26851826

[47] Beekman C, Sipkens JA, Testerink J, Giannakopoulos S, Kreuger D, van Deutekom JC, et al (2014) A sensitive, reproducible and objective immunofluorescence analysis method of dystrophin in individual fibers in samples from patients with duchenne muscular dystrophy. PLoS One 9: e107494 10.1371/journal.pone.0107494 25244123 PMC4171506

