# Supplementary figures and images for: Correlation of Utrophin Levels with the Dystrophin Protein Complex and Muscle Fibre Regeneration in Duchenne and Becker Muscular Dystrophy Muscle Biopsies
Source: PLoS One. 2016 Mar 14;11(3):e0150818. doi: 10.1371/journal.pone.0150818 (PMC4790853; doi:10.1371/journal.pone.0150818)

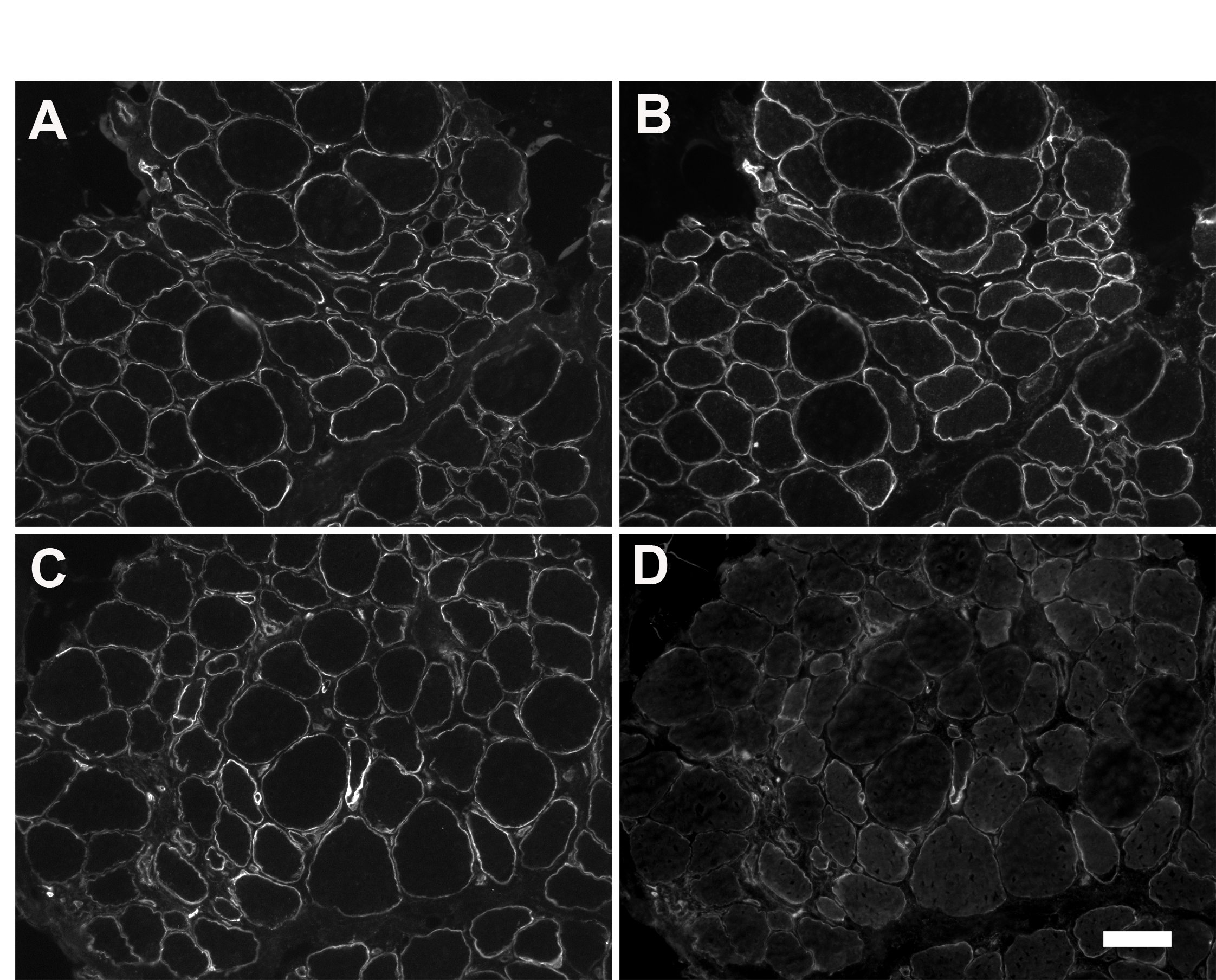

Supplement: S1 Fig — Scale bar = 50μM. (TIFF) [file pone.0150818.s001.tiff]

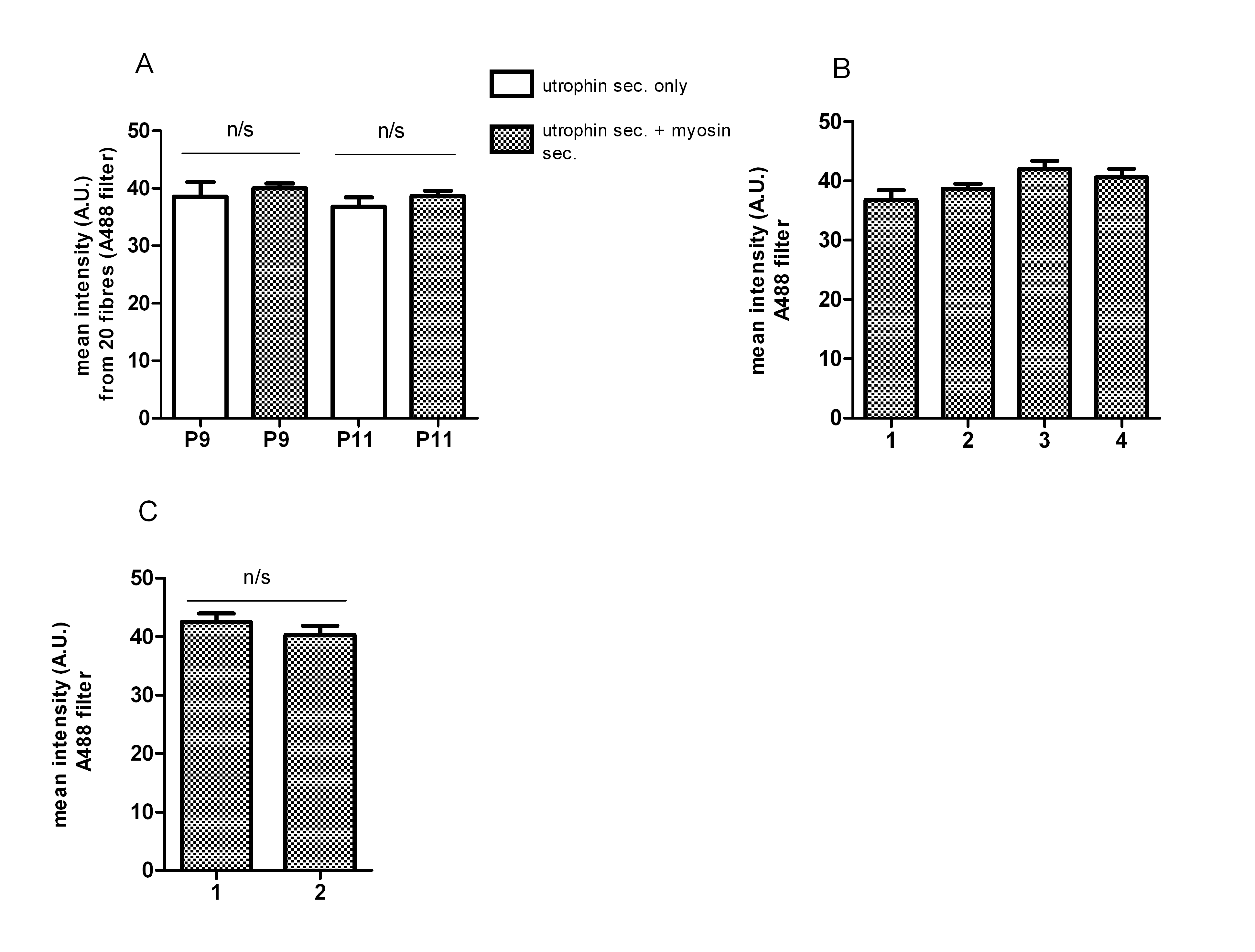

Supplement: S2 Fig — A. Serial sections of P9 and P11 were labelled with either the secondary antibody used for utrophin immunostaining only, or with a combination of both secondary antibodies used for fetal myosins and utrophin immunostaining. Images were captured for sarcolemmal intensity measurement in 20 random fibres, using the same exposure times used for utrophin intensity measurements. There was no difference in mean sarcolemmal intensity between the sections stained with one or both secondary antibodies for both P9 (unpaired t-test; p = 0.58) and P11 (unpaired t-test; p = 0.32). B. Serial sections of P11 were stained with either (1) Secondary antibody for utrophin only (2) secondary antibodies for utrophin and fetal myosins (3) secondary antibodies for utrophin and gamma sarcoglycan (g-SG) (4) secondary antibodies for utrophin and beta-dystroglycan (b-DG) and images captured for sarcolemmal intensity measurement. 1 and 2: 20 fibres analysed for each; 3 and 4: 10 fibres analysed for each. There were no differences in sarcolemmal intensity between the 4 groups (1 way ANOVA with Tukey’s test). C. Serial sections of P12 were stained with secondary antibodies for (1) utrophin and g-SG, or (2) utrophin and b-DG and images captured for sarcolemmal intensity analysis. 10 fibres were quantified for each combination. There was no significant difference between sarcolemmal intensity between the two groups (unpaired t-test; p = 0.32). The sarcolemmal intensities when only secondary antibodies had been applied were similar to the mean utrophin intensity levels in control muscles (38 ± 1.7; Fig 2). (TIFF) [file pone.0150818.s002.tiff]

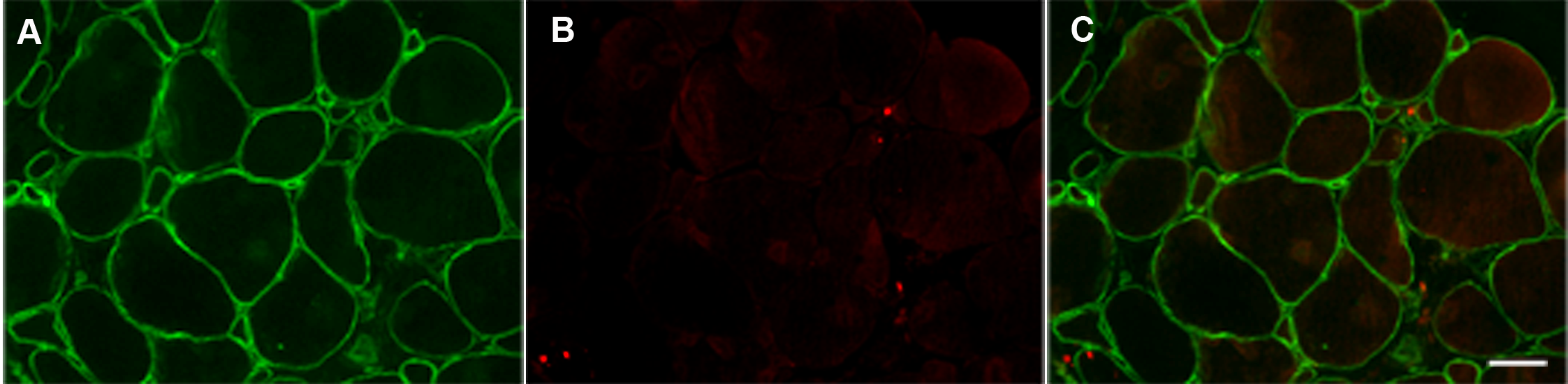

Supplement: S3 Fig — Section of P12 was stained with mouse monoclonal antibody to utrophin, (NCL-DRP2 IgG1), followed by Alexa Fluor 488 donkey anti mouse antibody (Molecular Probes A21202, anti IgG) and the secondary antibody that was used to detect beta-dystroglycan (Alexa Fluor A594 goat anti mouse IgG2A (Molecular Probes A21135). A. Fibres expressing utrophin, detected by Alexa Flour 488 donkey anti mouse IgG antibody. B. Alexa Fluor A594 goat anti mouse IgG2A antibody does not cross-react with utrophin. C. Merged image of A and B. Scale bar = 50μM. (TIFF) [file pone.0150818.s003.tiff]
