# Supplementary material for: Correlation of Utrophin Levels with the Dystrophin Protein Complex and Muscle Fibre Regeneration in Duchenne and Becker Muscular Dystrophy Muscle Biopsies
Source: PLoS One. 2016 Mar 14;11(3):e0150818. doi: 10.1371/journal.pone.0150818 (PMC4790853; doi:10.1371/journal.pone.0150818)
Supplement: S1 Table — (DOCX) [file pone.0150818.s004.docx]

**S1** Table. Sarcolemmal intensity measurements after application of secondary and tertiary antibodies.

| Sample | Secondary antibody | Total fibres analysed | Mean intensity (A.U.) –A488 filter | Standard Error of Mean |
| --- | --- | --- | --- | --- |
| P9 | Alexa Flour 488 donkey anti mouse | 20 | 38.5 | 2.5 |
| P9 | Alexa Flour 488 donkey anti mouse / biotinylated anti-mouse + streptavidin conjugated to Alexa Fluor 594 | 20 | 40 | 0.8 |
| P11 | Alexa Flour 488 donkey anti mouse | 20 | 36.8 | 1.7 |
| P11 | Alexa Flour 488 donkey anti mouse / biotinylated anti-mouse + streptavidin conjugated to Alexa Fluor 594 | 20 | 38.7 | 0.8 |
| P11 | Alexa Flour 488 donkey anti mouse / Alexa Fluor A594 goat anti rabbit IgG1 | 10 | 42 | 1.4 |
| P11 | Alexa Flour 488 donkey anti mouse / Alexa Fluor A594 goat anti mouse IgG2a | 10 | 40.6 | 1.4 |
| P12 | Alexa Flour 488 donkey anti mouse / Alexa Fluor A594 goat anti rabbit IgG1 | 10 | 42.5 | 1.5 |
| P12 | Alexa Flour 488 donkey anti mouse / Alexa Fluor A594 goat anti mouse IgG2a | 10 | 40.3 | 1.6 |
